# Identification of high-performing antibodies for tyrosine-protein kinase SYK for use in Western Blot, immunoprecipitation and immunofluorescence

**DOI:** 10.12688/f1000research.140456.1

**Published:** 2023-09-27

**Authors:** Walaa Alshafie, Maryam Fotouhi, Riham Ayoubi, Kathleen Southern, Carl Laflamme, NeuroSGC/YCharOS collaborative group

**Affiliations:** 1 Department of Neurology and Neurosurgery, Structural Genomics Consortium, The Montreal Neurological Institute, McGill University, Montreal, Québec, H3A 2B4, Canada

**Keywords:** Uniprot ID P43405, SYK, tyrosine-protein kinase SYK, spleen tyrosine kinase, antibody characterization, antibody validation, Western Blot, immunoprecipitation

## Abstract

Tyrosine-protein kinase SYK, encoded by the *SYK* gene, is a non-receptor type protein kinase which mediates immune signal transduction through immunoreceptors. Tyrosine-protein kinase SYK expression has been associated with the development of various inflammatory diseases, cancer and neurodegenerative conditions. The reproducibility of tyrosine-protein kinase SYK research would help elucidate the mechanism in which it causes neuroinflammation as well as its potential as a novel target to treat Alzheimer’s disease. This would be facilitated with the availability of high-quality tyrosine-protein kinase SYK. In this study, we characterized thirteen tyrosine-protein kinase SYK commercial antibodies for Western Blot, immunoprecipitation, and immunofluorescence using a standardized experimental protocol based on comparing read-outs in knockout cell lines and isogenic parental controls. We identified many high-performing antibodies and encourage readers to use this report as a guide to select the most appropriate antibody for their specific needs.

## Introduction

Tyrosine-protein kinase SYK, also known as spleen tyrosine kinase (SYK), is a non-receptor type of protein-tyrosine kinase (PTK) predominantly recognized for its role in amplifying immune responses. ^1^ Unique to other families of PTKs, SYK has tandem N-terminal Src homology 2 (SH2) domains as well as a tyrosine kinase domain at its C-terminal region. Binding of the SH2 domains to di-phosphorylated immunoreceptor tyrosine-based activating motif (ITAM) activates SYK, triggering downstream inflammatory signalling cascades. ^1^^–^^3^

Functioning as a vital mediator of cellular inflammatory responses, SYK is primarily known to contribute to allergies, ^4^ autoimmune diseases ^5^^,^^6^ and B-cell malignancies. ^7^ Emerging research has suggested SYK may be implicated in the development in neuroinflammatory symptoms that are characteristic of Alzheimer’s disease (AD). ^8^^,^^9^ As such, studies have demonstrated that inhibition or down-regulation of SYK increases amyloid-beta (Aβ) clearance and decreases Tau hyperphosphorylation, highlighting it’s potential as a therapeutic target to treat AD. ^9^^–^^11^ Further investigation is required to elucidate the mechanism in which SYK influences Tau pathology and Aβ deposition. ^8^

Mechanistic studies would be greatly facilitated with the availability of high-quality antibodies. Here, we compared the performance of a range of commercially available antibodies for tyrosine-protein kinase SYK and validated several antibodies for Western Blot, immunoprecipitation and immunofluorescence, enabling biochemical and cellular assessment of tyrosine-protein kinase SYK properties and function.

## Results and discussion

Our standard protocol involves comparing readouts from wild-type and knockout cells. ^12^^–^^22^ To identify a cell line that expresses adequate levels of tyrosine-protein kinase SYK protein expression to provide sufficient signal to noise, we examined public proteomics databases, namely PaxDB ^23^ and DepMap. ^24^ THP-1 was identified as a suitable cell line and thus THP-1 was modified with CRISPR/Cas9 to knockout the corresponding *SYK* gene ( Table 1).

**Table 1.** Summary of the cell lines used.

Institution	Catalog number	RRID (Cellosaurus)	Cell line	Genotype
Abcam	ab271147	CVCL_0006	THP-1	WT
Abcam	ab288700	-	THP-1	*SYK* KO

**Figure 1.**
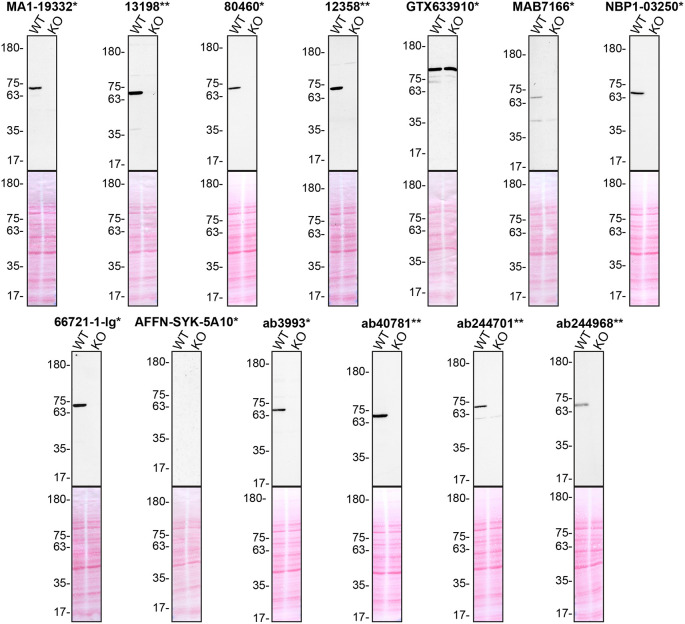
Tyrosine-protein kinase SYK antibody screening by Western Blot. Lysates of THP-1 (WT and *SYK* KO) were prepared, and 30 μg of protein were processed for Western Blot with the indicated tyrosine-protein kinase SYK antibodies. The Ponceau stained transfers of each blot are presented to show equal loading of WT and KO lysates and protein transfer efficiency from the acrylamide gels to the nitrocellulose membrane. Antibody dilutions were chosen according to the recommendations of the antibody supplier. An exception was given for antibody GTX633910*, which was titrated to 1/250 as the signal was too weak when following the supplier’s recommendations. Antibody dilutions used: MA1-19332* at 1/1000, 13198** at 1/1000, 80460* at 1/1000, 12358** at 1/1000, GTX633910* at 1/250, MAB7166* at 1/250, NBP1-03250* at 1/500, 66721-1-lg* at 1/2000, AFFN-SYK-5A10* at 1/200, ab3993* at 1/500, ab40781** at 1/1000, ab244701** at 1/1000, ab244968** at 1/1000. Predicted band size: 72 kDa. *Monoclonal antibody, **Recombinant antibody.

For Western Blot experiments, we resolved proteins from WT and *SYK* KO cell extracts and probed them side-by-side with all antibodies in parallel ^13^^–^^22^ ( Figure 1).

**Figure 2.**
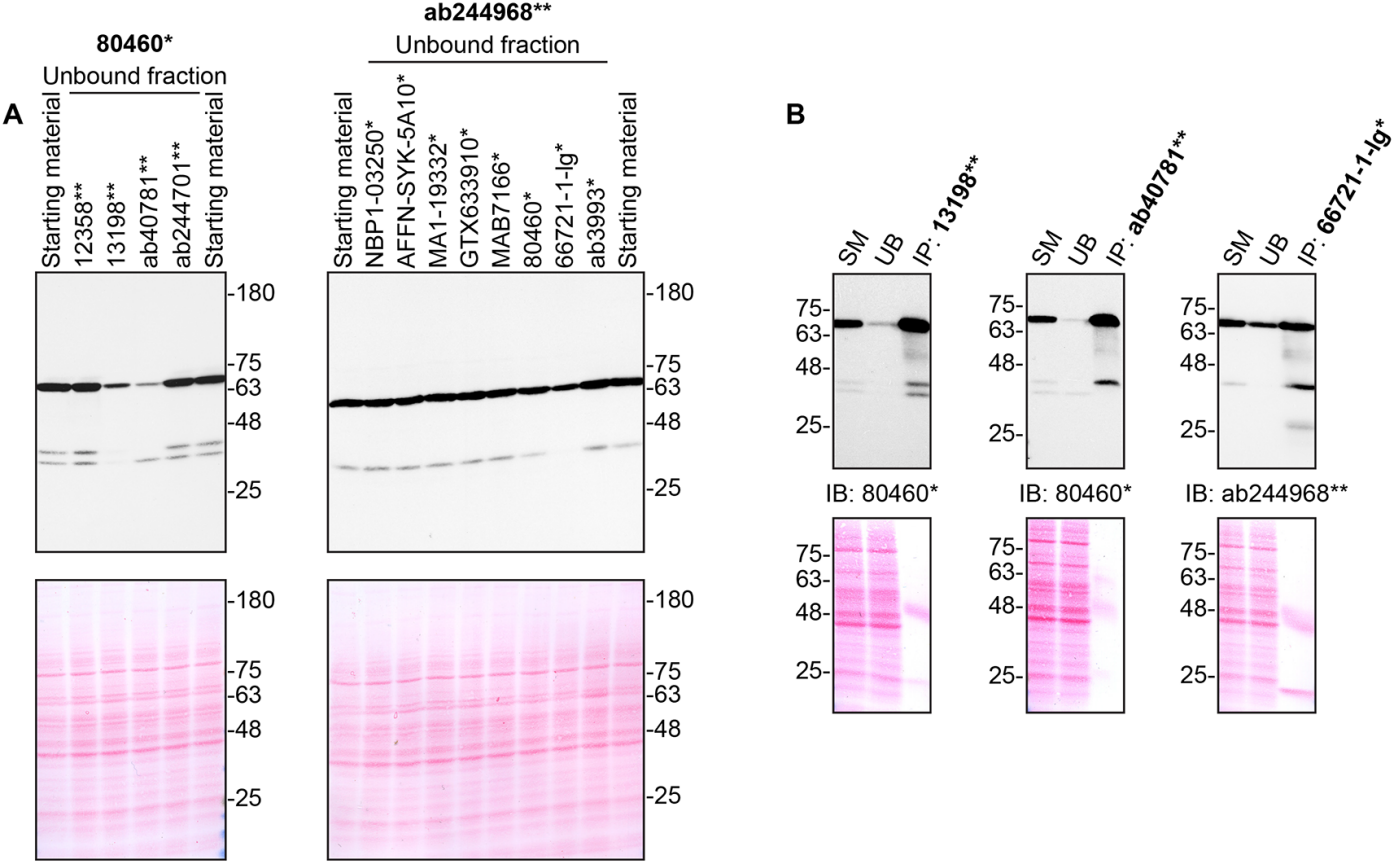
Tyrosine-protein kinase SYK antibody screening by immunoprecipitation. THP-1 lysates were prepared, and immunoprecipitation was performed using 2.0 μg of the indicated tyrosine-protein kinase SYK antibodies pre-coupled to Dynabeads protein G or protein A. A) Ability of the antibodies to immunocapture tyrosine-protein kinase SYK was first assessed by comparing the level of protein available in the starting material to the level remaining in the unbound fractions. B) The immunoprecipitates for antibodies which would immunocapture tyrosine-protein kinase SYK in (A) are shown. For Western Blot, 80460* and ab244968** were used at 1/3000. The Ponceau stained transfers of each blot are shown. SM = 10% starting material; UB = 10% unbound fraction; IP = immunoprecipitated. *Monoclonal antibody, **Recombinant antibody.

For immunoprecipitation experiments, we used the antibodies to immunopurify tyrosine-protein kinase SYK from THP-1 cell extracts. The performance of each antibody was first evaluated by detecting whether they could immunocapture tyrosine-protein kinase SYK. Antibodies that successfully captured the protein were further evaluated by immunoprecipitation ^13^^–^^22^ ( Figure 2).

**Figure 3.**
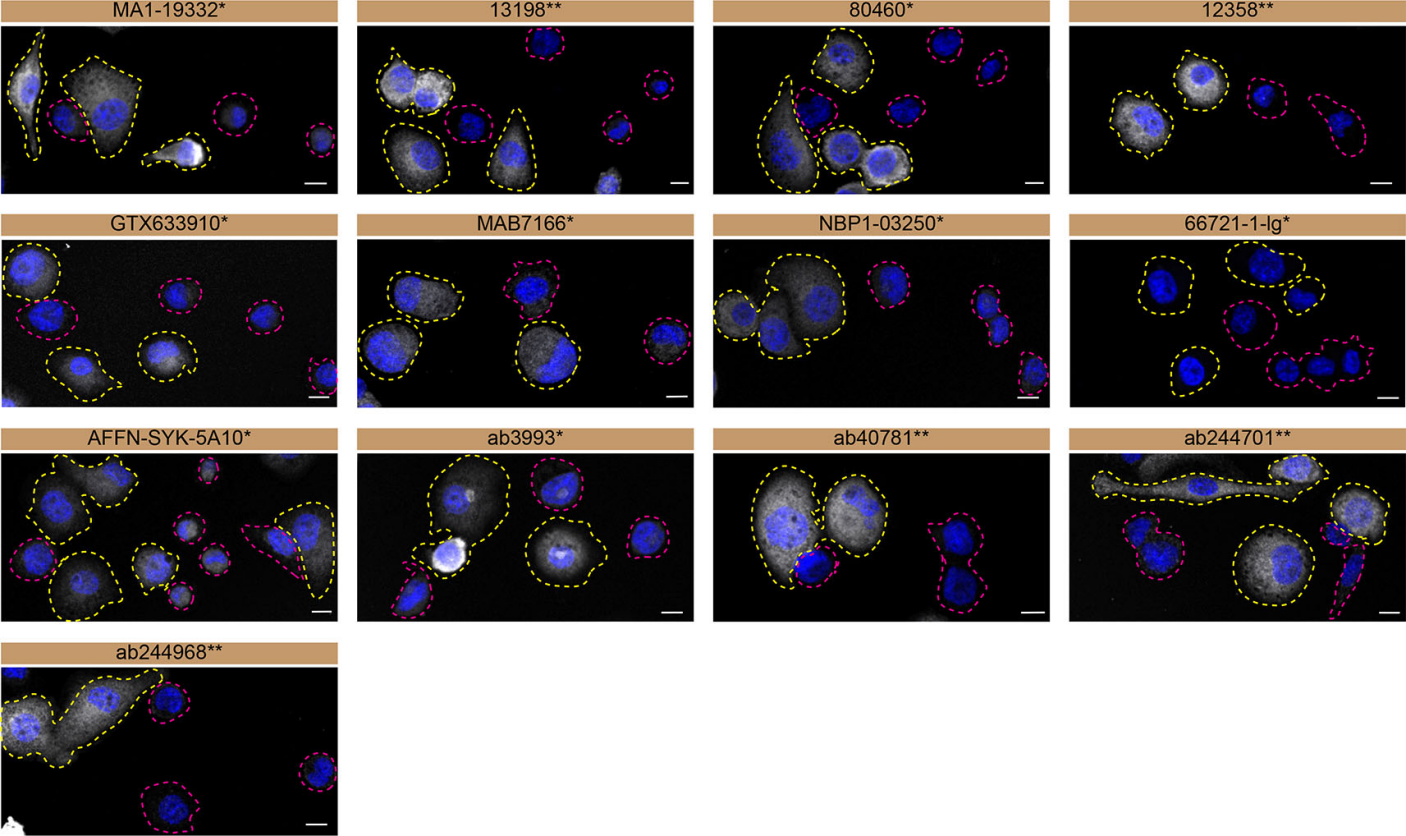
Tyrosine-protein kinase SYK antibody screening by immunofluorescence. THP-1 WT and *SYK* KO cells were labelled with a green or a far-red fluorescent dye, respectively. WT and KO cells were mixed and plated to a 1:1 ratio on coverslips. Cells were stained with the indicated tyrosine-protein kinase SYK antibodies and with the corresponding Alexa-fluor 555 coupled secondary antibody including DAPI. Acquisition of the blue (nucleus-DAPI), green (WT), red (antibody staining) and far-red (KO) channels was performed. Representative images of the merged blue and red (grayscale) channels are shown. WT and KO cells are outlined with yellow and magenta dashed line, respectively. Antibody dilutions were chosen according to the recommendations of the antibody supplier. Exceptions were given for antibodies GTX633910* and 66721-1-lg*, which were titrated to 1/2000 and 1/1000, respectively, as the signals were too weak when following the supplier’s recommendations. When the concentration was not indicated by the supplier, which was the case for antibodies ab3993*, ab244701**, and ab244968**, we tested antibodies at 1/1000. At this concentration, the signal from each antibody was in the range of detection of the microscope used. Antibody dilution used: MA1-19332* at 1/1000, 13198** at 1/400, 80460* at 1/400, 12358** at 1/400, GTX633910* at 1/2000, MAB7166* at 1/500, NBP1-03250* at 1/500, 66721-1-lg* at 1/1000, AFFN-SYK-5A10* at 1/60, ab3993* at 1/1000, ab40781** at 1/700, ab244701** at 1/1000, ab244968** at 1/1000. Bars = 10 μm. *Monoclonal antibody, **Recombinant antibody.

For immunofluorescence, as described previously, antibodies were screened using a mosaic strategy. ^25^ In brief, we plated WT and KO cells together in the same well and imaged both cell types in the same field of view to reduce staining, imaging and image analysis bias ( Figure 3).

In conclusion, we have screened tyrosine-protein kinase SYK commercial antibodies by Western Blot, immunoprecipitation and immunofluorescence and identified several high-quality antibodies under our standardized experimental conditions. Under our standardized experimental conditions, several high-quality antibodies were identified, however, the authors do not engage in result analysis or offer explicit antibody recommendations. A limitation of this study is the use of universal protocols - any conclusions remain relevant within the confines of the experimental setup and cell line used in this study. Our primary aim is to deliver top-tier data to the scientific community, grounded in Open Science principles. This empowers experts to interpret the characterization data independently, enabling them to make informed choices regarding the most suitable antibodies for their specific experimental needs. The underlying data supporting this study is found on Zenodo, an open access repository. ^26^^,^^27^

## Methods

### Antibodies

All tyrosine-protein kinase SYK antibodies are listed in Table 2, together with their corresponding Research Resource Identifiers, or RRID, to ensure the antibodies are cited properly. ^28^ Peroxidase-conjugated goat anti-rabbit and anti-mouse antibodies are from Thermo Fisher Scientific (cat. number 65-6120 and 62-6520). Alexa-555-conjugated goat anti-rabbit and anti-mouse secondary antibodies are from Thermo Fisher Scientific (cat. number A21429 and A21424).

**Table 2.** Summary of the Tyrosine-protein kinase SYK antibodies tested.

Company	Catalog number	Lot number	RRID (Antibody Registry)	Clonality	Clone ID	Host	Concentration (μg/μL)	Vendors recommended applications
Thermo Fisher Scientific	MA1-19332 *	Wb3186395	AB_2197214	monoclonal	SYK-01	mouse	1.00	Wb, IP, IF
Cell Signaling Technology	13198 **	9	AB_2687924	recombinant-mono	D3Z1E	rabbit	not provided	Wb, IP
Cell Signaling Technology	80460 *	1	AB_2799953	monoclonal	4D10	mouse	not provided	Wb, IP, IF
Cell Signaling Technology	12358 **	1	AB_2687923	recombinant-mono	D1l5Q	rabbit	not provided	Wb, IP
GeneTex	GTX633910 *	43314	AB_2888388	monoclonal	GT351	mouse	2.83	Wb, IF
Bio-Techne	MAB7166 *	CFUQ0117021	AB_10972948	monoclonal	720402	mouse	0.50	Wb
Bio-Techne	NBP1-03250 *	199549	AB_1522471	monoclonal	SYK-01	mouse	0.50	Wb, IP
Proteintech	66721-1-lg *	10006710	AB_2882072	monoclonal	4C4A12	mouse	1.00	Wb, IF
Developmental Studies Hybridoma Bank	AFFN-SYK-5A10 *	4/28/2016	AB_2617957	monoclonal	AFFN-SYK-5A10	mouse	0.062	other
Abcam	ab3993 *	GR3203808-21	AB_304217	monoclonal	SYK-01	mouse	1.00	Wb, IF
Abcam	ab40781 **	GR3273231-3	AB_778196	recombinant-mono	EP573Y	rabbit	0.71	Wb, IF
Abcam	ab244701 **	GR3273514-3	AB_2910244 ^1^	recombinant-mono	EPR19414-176	rabbit	1.01	other
Abcam	ab244968 **	GR3273515-3	AB_2910245 ^1^	recombinant-mono	EPR573-69	rabbit	0.99	other

Wb = Western Blot; IF = immunofluorescence; IP = immunoprecipitation.

* Monoclonal antibody.

** Recombinant antibody.

^1^ Refers to RRID that were recently created (July 2023) and will be added to the Antibody Registry in the coming weeks.

### CRISPR/Cas9 genome editing

Cell lines used are listed in Table 1. THP-1 *SYK* KO clone was generated with low passage cells using an open-access protocol available on Zenodo.org: https://zenodo.org/record/3875777#.ZA-Rxi-96Rv. Two guide RNAs were used to knockout *SYK* in THP-1 using the CRISPR-Cas9 technology (sequence guide 1: TTTCGGCAACATCACCCGGG, sequence guide 2: GCTCCCGCTCGATGGTGTAG).

### Cell culture

Both THP-1 WT and *SYK* KO cell lines used are listed in Table 1, together with their corresponding RRID, to ensure the cell lines are cited properly. ^29^ Cells were cultured in DMEM high-glucose (GE Healthcare cat. number SH30081.01) containing 10% fetal bovine serum (Wisent, cat. number 080450), 2 mM L-glutamate (Wisent cat. number 609065, 100 IU penicillin and 100 μg/mL streptomycin (Wisent cat. number 450201).

### Antibody screening by Western Blot

Western Blots were performed as described in our standard operating procedure. ^30^ THP-1 WT and *SYK* KO were collected in RIPA buffer (25 mM Tris-HCl pH 7.6, 150 mM NaCl, 1% NP-40, 1% sodium deoxycholate, 0.1% SDS) (Thermo Fisher Scientific, cat. number 89901) supplemented with protease inhibitor. Lysates were sonicated briefly and incubated for 30 min on ice. Lysates were spun at ~110,000 × g for 15 min at 4°C and equal protein aliquots of the supernatants were analyzed by SDS-PAGE and Western Blot. BLUelf prestained protein ladder (GeneDireX, cat. number PM008-0500) was used.

Western Blots were performed with large 5–16% gradient polyacrylamide gels and transferred on nitrocellulose membranes. Proteins on the Blots were visualized with Ponceau S staining (Thermo Fisher Scientific, cat. number BP103-10) which is scanned to show together with individual Western Blots. Blots were blocked with 5% milk for 1 hr, and antibodies were incubated overnight at 4°C with 5% bovine serum albumin (BSA) (Wisent, cat. number 800-095) in TBS with 0.1% Tween 20 (TBST) (Cell Signalling Technology, cat. number 9997). Following three washes with TBST, the peroxidase conjugated secondary antibody was incubated at a dilution of ~0.2 μg/mL in TBST with 5% milk for 1 hr at room temperature followed by three washes with TBST. Membranes were incubated with Pierce ECL (Thermo Fisher Scientific, cat. number 32106) prior to detection with the HyBlot CL autoradiography films (Denville, cat. number 1159T41).

### Antibody screening by immunoprecipitation

Immunoprecipitation was performed as described in our standard operating procedure. ^31^ Antibody-bead conjugates were prepared by adding 2 μg or 10 μL of antibody at an unknown concentration to 500 μL of Pierce IP Lysis Buffer (Thermo Fisher Scientific, cat. number 87788) in a 1.5 mL microcentrifuge tube, together with 30 μL of Dynabeads protein A - (for rabbit antibodies) or protein G - (for mouse antibodies) (Thermo Fisher Scientific, cat. number 10002D and 10004D). Tubes were rocked for ~ 2 hrs at 4°C followed by several washes to remove unbound antibodies.

THP-1 WT were collected in Pierce IP buffer (25 mM Tris-HCl pH 7.4, 150 mM NaCl, 1 mM EDTA, 1% NP-40 and 5% glycerol) supplemented with protease inhibitor. Lysates were rocked 30 min at 4°C and spun at 110,000 × g for 15 min at 4°C. One mL aliquots at 1.0 mg/mL of lysate were incubated with an antibody-bead conjugate for ~2 hrs at 4°C. The unbound fractions were collected, and beads were subsequently washed three times with 1.0 mL of IP lysis buffer and processed for SDS-PAGE and Western Blot on 5–16% gradient polyacrylamide gels.

### Antibody screening by immunofluorescence

Immunofluorescence was performed as described in our standard operating procedure. ^13^^–^^22^^,^^25^ THP-1 WT and *SYK* KO were labelled with a green and a far-red fluorescence dye, respectively (Thermo Fisher Scientific, cat. number C2925 and C34565). The nuclei were labelled with DAPI (Thermo Fisher Scientific, cat. number D3571) fluorescent stain. WT and KO cells were plated on glass coverslips as a mosaic and incubated for 24 hrs in a cell culture incubator at 37 ^o^C, 5% CO _2_. Cells were fixed in 4% paraformaldehyde (PFA) (Beantown chemical, cat. number 140770-10 ml) in phosphate buffered saline (PBS) (Wisent, cat. number 311-010-CL). Cells were permeabilized in PBS with 0,1% Triton X-100 (Thermo Fisher Scientific, cat. number BP151-500) for 10 min at room temperature and blocked with PBS with 5% BSA, 5% goat serum (Gibco, cat. number 16210-064) and 0.01% Triton X-100 for 30 min at room temperature. Cells were incubated with IF buffer (PBS, 5% BSA, 0,01% Triton X-100) containing the primary tyrosine-protein kinase SYK antibodies overnight at 4°C. Cells were then washed 3 × 10 min with IF buffer and incubated with corresponding Alexa Fluor 555-conjugated secondary antibodies in IF buffer at a dilution of 1.0 μg/mL for 1 hr at room temperature with DAPI. Cells were washed 3 × 10 min with IF buffer and once with PBS. Coverslips were mounted on a microscopic slide using fluorescence mounting media (DAKO).

Imaging was performed using a Zeiss LSM 700 laser scanning confocal microscope equipped with a Plan-Apo 20x air objective (NA = 0.8). All cell images represent a single focal plane. Figures were assembled with Adobe Photoshop (version 24.1.2) to adjust contrast then assembled with Adobe Illustrator (version 27.3.1).

## Data Availability

Zenodo: Antibody Characterization Report for tyrosine-protein kinase SYK, https://doi.org/10.5281/zenodo.6566940. ^26^ Zenodo: Dataset for the tyrosine-protein kinase SYK antibody screening study, https://doi.org/10.5281/zenodo.8164709. ^27^ Data are available under the terms of the Creative Commons Attribution 4.0 International license (CC-BY 4.0)
