# Supplementary material for: Identification of high-performing antibodies for tyrosine-protein kinase SYK for use in Western Blot, immunoprecipitation and immunofluorescence
Source: F1000Res. 2024 Jun 11;12:1222. Originally published 2023 Sep 27. [Version 3] doi: 10.12688/f1000research.140456.3 (PMC11214040; doi:10.12688/f1000research.140456.3)
Supplement: Peer review history

# Reviewer report: Reviewer response for version 3

**DOI:** 10.5256/f1000research.167657.r289607

**Published:** 2024-06-28

**Authors:** Araujo Nelson A

**Affiliations:** 1 Universidad de O'Higgins, Rancagua, O'Higgins Region, Chile

The answers to the questions presented by the authors were satisfactory.

Are sufficient details of methods and materials provided to allow replication by others?

Yes

Is the rationale for creating the dataset(s) clearly described?

Yes

Are the datasets clearly presented in a useable and accessible format?

Yes

Are the protocols appropriate and is the work technically sound?

Yes

Reviewer Expertise:

Chemistry of Proteins and Peptides

I confirm that I have read this submission and believe that I have an appropriate level of expertise to confirm that it is of an acceptable scientific standard.

# Reviewer report: Reviewer response for version 2

**DOI:** 10.5256/f1000research.157120.r235832

**Published:** 2024-02-16

**Authors:** Araujo Nelson A

**Affiliations:** 1 Universidad de O'Higgins, Rancagua, O'Higgins Region, Chile

Full Report.

- The data note entitled "Identification of high-performing antibodies for tyrosine-protein kinase SYK for use in Western Blot, immunoprecipitation, and immunofluorescence" compares thirteen commercial antibodies against tyrosine-protein kinase SYK using three different immunodetection techniques to provide relevant data regarding the sensitivity and specificity of these antibodies for SYK protein tyrosine kinase.
- My main criticism is that the results and discussion section is little developed; for example, are not briefly described the most relevant results of any of the three experiments and are not discussed the results that were different, for example, why the GTX633910 antibody detects a polypeptide band above 75 kDa while the 72 kDa band corresponding to the Tyrosine-protein kinase SYK is very poorly detected; why  is not detected with the AFFN-SYK-5A10 antibody the  Tyrosine-protein kinase SYK; why in immunoprecipitation the 80460 and ab244968 antibodies were  used  to immunodetect the unbound fraction and not other antibodies. The authors should provide answers in the text to these questions.
- In immunofluorescence results, place the white light micrograph of the cells next to the fluorescence image to observe the cell outline is recommended and can then be followed by the colored segmented lines in the fluorescence image.
- Write in the protocol how the total protein concentration in the supernatant was determined and how many  the volume in microliters used to obtain 30 mg of protein from WT cells as from SYK KO cells in each lane  to do better reproducible the experiment.
- Also the conclusion is observed underdeveloped. I recommend the authors to conclude by offering their own recommendations on bioanalytical applications of the antibodies analyzed and compare with the vendor-recommended applications read in Table 2.

Are sufficient details of methods and materials provided to allow replication by others?

Yes

Is the rationale for creating the dataset(s) clearly described?

Yes

Are the datasets clearly presented in a useable and accessible format?

Yes

Are the protocols appropriate and is the work technically sound?

Yes

Reviewer Expertise:

Structure and function of protein kinases A

I confirm that I have read this submission and believe that I have an appropriate level of expertise to confirm that it is of an acceptable scientific standard, however I have significant reservations, as outlined above.

# Response

**Published:** 2024-06-06

**Authors:** Kathleen Southern

**Affiliations:** McGill University, Canada

Thank you Araujo Nelson A. for your comprehensive reviewer report for our article. Please see below our responses to your specific comments and concerns.

- *The data note entitled "Identification of high-performing antibodies for tyrosine-protein kinase SYK for use in Western Blot, immunoprecipitation, and immunofluorescence" compares thirteen commercial antibodies against tyrosine-protein kinase SYK using three different immunodetection techniques to provide relevant data regarding the sensitivity and specificity of these antibodies for SYK protein tyrosine kinase.*
- *My main criticism is that the results and discussion section is little developed; for example, are not briefly described the most relevant results of any of the three experiments and are not discussed the results that were different, for example, why the GTX633910 antibody detects a polypeptide band above 75 kDa while the 72 kDa band corresponding to the Tyrosine-protein kinase SYK is very poorly detected; why  is not detected with the AFFN-SYK-5A10 antibody the  Tyrosine-protein kinase SYK; why in immunoprecipitation the 80460 and ab244968 antibodies were  used  to immunodetect the unbound fraction and not other antibodies. The authors should provide answers in the text to these questions.*

While the authors understand your concern regarding the lack of result analysis, we must re-iterate, that the authors do not engage in result analysis nor offer explicit antibody recommendations. The format of this article is a Data Note, with the goal being to provide brief descriptions of research data for potential reuse or to benefit for the scientific community without providing any analyses or conclusions, as explained in their article guidelines ( https://f1000research.com/for-authors/article-guidelines/data-notes).

 The GTX633910 antibody detecting bands above the 75 kDa molecular marker in both the wild-type (WT) and the knockout (KO) could indicate that this antibody is non-selective, binding to epitopes other than the intended target. The Western blot assessment of antibodies in the unbound fraction as well as the immunoprecipitation are carried out with antibodies previously KO-validated by Western blot (Figure 1), which is why antibodies 8060 and ab244968 were selected. We understand that this last point was not made clear in the text. In the newly submitted version of this article we have included a statement in the results and discussion section to clarify this part of the protocol.

- *In immunofluorescence results, place the white light micrograph of the cells next to the fluorescence image to observe the cell outline is recommended and can then be followed by the colored segmented lines in the fluorescence image.*

With our immunofluorescence protocol described in the methods section of this article, the goal is not to define the cellular location of the target protein but rather to determine where or not ratio of signal between the WT and KO cells is significantly different. This is why we outline of the cell is not as significant compared to the signal produced by the cells themselves.

- *Write in the protocol how the total protein concentration in the supernatant was determined and how many  the volume in microliters used to obtain 30 mg of protein from WT cells as from SYK KO cells in each lane  to do better reproducible the experiment.*

The protocol describing how the total protein concentration was determined as well as the method to obtain 30 mg of protein has been included in the Methods section describing Antibody screening by Western blot.

- *Also the conclusion is observed underdeveloped. I recommend the authors to conclude by offering their own recommendations on bioanalytical applications of the antibodies analyzed and compare with the vendor-recommended applications read in Table 2.*

The authors to not offer their own recommendations nor compare the antibody performance in each application to the vendors recommended application. The reason being that the antibodies are tested under one specific set of conditions, therefore summarizing the performance of the antibodies would only be valid under these precise experimental set-up and cell lines used. The goal of the YCharOS initiative is to present antibody characterization data to the scientific community, using a standardized protocol that allows researchers to select high-performing antibodies. This enables researchers who specialize in the target of interest, in this case Tyrosine -protein kinase SYK, to conduct further studies. This intention is clearly defined in the concluding paragraph of the results and discussion section.

 We hope that with our clarifications, responses to your concerns, and the statements included in the latest version of the article, you no longer have reservations about the status of this article. The presented antibody characterization data will be highly beneficial for researchers aiming to enhance their studies on tyrosine-protein kinase SYK.

# Reviewer report: Reviewer response for version 2

**DOI:** 10.5256/f1000research.157120.r228375

**Published:** 2024-01-03

**Authors:** Kiyonao Sada

**Affiliations:** 1 University of Fukui, Fukui, Japan

The importance of the protein-tyrosine kinase Syk has been highlighted in new research areas for studying the pathogenesis and developing treatments of neurological diseases. In this study, the authors compare the usefulness of anti-Syk antibody-based approaches using wild-type and Syk-deficient THP-1 cells, established by genome editing.

 It is very important to know which antibodies commercially available are useful for a particular protein, not just Syk. This paper does not indicate which antibodies are recommended, but allows experts reading this paper to make their own interpretations. The experimental conditions are based on the recommended dilution factors, and in practice, further investigation by the researcher is required. However, it is not practical for many researchers to purchase and compare all antibodies, so the Syk results provide very important information.

 Western blot results were relatively uniform, but differences in the usefulness of each antibody were observed in immunoprecipitation and immunofluorescence. In particular, the reader is provided with the information that some antibodies could detect Syk effectively despite the lack of company recommendations for immunofluorescence.

 Regarding Table 2, in discussing the experimental results, it is also necessary to present information about the antigens of these antibodies. This is because it is assumed that when immunoprecipitation is performed, the antibodies mask the antigenic sites, making it difficult to detect proteins that associate with the same sites.

 Overall, I can conclude that this is a very useful paper for researchers who are pioneering new studies on Syk.

Are sufficient details of methods and materials provided to allow replication by others?

Yes

Is the rationale for creating the dataset(s) clearly described?

Yes

Are the datasets clearly presented in a useable and accessible format?

Partly

Are the protocols appropriate and is the work technically sound?

Yes

Reviewer Expertise:

Signal transduction

I confirm that I have read this submission and believe that I have an appropriate level of expertise to confirm that it is of an acceptable scientific standard.
